# Plasma glutamine levels before cardiac surgery are related to post-surgery infections; an observational study

**DOI:** 10.1186/s13019-016-0549-1

**Published:** 2016-11-25

**Authors:** Hanneke Buter, Matty Koopmans, Ramses Kemperman, Lilian Jekel, Christiaan Boerma

**Affiliations:** 1 Departments of Intensive Care, Medical Centre Leeuwarden, Henri Dunantweg 2, 8935 AD Leeuwarden, The Netherlands; 2 Clinical Chemistry, Medical Centre Leeuwarden, Leeuwarden, The Netherlands; 3 Cardiovascular Surgery, Medical Centre Leeuwarden, Leeuwarden, The Netherlands

**Keywords:** Plasma glutamine level, Cardiac surgery, Postoperative infection

## Abstract

**Background:**

A low plasma glutamine level was found in 34% of patients after elective cardiothoracic surgery. This could be a result of the inflammation caused by surgical stress or the use of extracorporeal circulation (ECC). But it is also possible that plasma glutamine levels were already lowered before surgery and reflect an impaired metabolic state and a higher likelihood to develop complications.

In the present study plasma glutamine levels were measured before and after cardiac surgery and we questioned whether there is a relation between plasma glutamine levels and duration of ECC and the occurrence of postoperative infections.

**Methods:**

We performed a single-centre prospective, observational study in a closed-format, 20-bed, mixed ICU in a tertiary teaching hospital. We included consecutive patients after elective cardiac surgery with use of extracorporeal circulation. Blood samples were collected on the day prior to surgery and at admission on the ICU.

The study was approved by the local Medical Ethics Committee (Regional Review Committee Patient-related Research, Medical Centre Leeuwarden, nWMO 115, April 28th 2015).

**Results:**

Ninety patients were included. Pre-operative plasma glutamine level was 0.42 ± 0.10 mmol/l and post-operative 0.38 ± 0.09 mmol/l (*p* < 0.001). There was no relation between duration of extracorporeal circulation or aortic occlusion time and changes in plasma glutamine levels. A logistic regression analysis showed a significant correlation between the presence of a positive culture during the post-operative course and pre-operative plasma glutamine levels (*p* = 0.04).

**Conclusion:**

Plasma glutamine levels are significantly lower just after cardiac surgery compared to pre-operative levels. We did not find a relation between the decrease in plasma glutamine levels and the duration of extracorporeal circulation or aortic clamp time. There was a correlation between pre-operative plasma glutamine levels and the presence of a positive culture after cardiac surgery.

**Trial registration:**

ClinicalTrials.gov, number NCT02444780.

## Background

Low plasma glutamine levels at the time of admission on the Intensive Care Unit are associated with an unfavourable outcome [1, 2]. The mechanism behind the association of a low plasma glutamine level with severity of illness or mortality in critical illness is not fully understood [3]. Glutamine, which is released from muscle cells, is an important nutrient during periods of metabolic stress, especially for immune cells and enterocytes [4–6].

There is an association between low plasma glutamine levels and the presence of an infection at the time of admission on the ICU [7]. These low plasma glutamine levels could be the result of the inflammatory response in reaction to an infection but it is also possible that plasma glutamine levels were already low in these patients and reflect a metabolic state which makes patients more vulnerable to develop an infection.

In 34% of patients who were admitted to our ICU after elective cardiac surgery a low plasma glutamine level was found [7]. This could be result of the inflammation caused by surgical stress or the use of extracorporeal circulation (ECC) [8, 9]. But it is also possible that plasma glutamine levels were already lowered before surgery in some of these patients and reflects an impaired metabolic state and a higher likelihood to develop complications.

In the present study we measured plasma glutamine levels before and after cardiac surgery and questioned whether there is a relation between plasma glutamine levels and duration of ECC and the occurrence of postoperative infections.

## Methods

### Study design

We performed a single-centre prospective, observational study in a closed-format, 20-bed, mixed ICU in a tertiary teaching hospital. We included 90 consecutive patients who were admitted to the ICU in June until August 2015 after elective cardiac surgery with use of extracorporeal circulation. Patients had to be older than 18 years, there were no further exclusion criteria.

The study was approved by the local Medical Ethics Committee (Regional Review Committee Patient-related Research, Medical Centre Leeuwarden, nWMO 115, April 28th 2015) and has been performed in accordance with the ethical standard laid down in the 1964 Declaration of Helsinki and its later amendments. Informed consent was waived under the condition that the plasma glutamine measurements were performed from residue blood samples, taken as part of standard care according to protocol. The study was registered in a public register (ClinicalTrials.gov, number NCT02444780).

### Setting

Blood samples were collected when patients were admitted to the hospital, the day prior to surgery. According to protocol the pre-operative plasma glutamine level was determined from residue blood samples. At admission on the ICU blood samples were collected according to protocol, from residue blood the post-operative plasma glutamine level was determined.

All patients received a total of 4 doses of cefazolin 1000 mg in 24 h, starting 30 min prior to surgery to prevent postoperative infections. Furthermore, all patients used Bactroban® (Mupirocine) 3 times daily during 3 days to eradicate staphylococcus aureus from the nose cavity, starting on the day prior to surgery.

### Glutamine measurements

Measurement of plasma glutamine level was performed with the Bioprofile 100 plus analyser (Nova Biomedical U.K., Cheshire, UK). This analyser was originally intended for use for monitoring metabolites and nutrients in cell culture systems, and was used in this study for investigational purposes to monitor glutamine levels in plasma samples [7]. A low plasma glutamine level is defined as a concentration < 420 mmol/l. This is the lower limit of glutamine as suggested by the New England Journal of Medicine [8].

### Statistical analysis

In our previous study 34% of patients who were admitted to the ICU after elective surgery had a plasma glutamine level of 0.42 mmol/l or less [7]. In the present study we aimed to include about 30 patients with a low plasma glutamine level so we set the number of patients to include in this study on 90 patients.

Data are shown as the mean with standard deviation or median with IQR, according to their distribution. The difference between pre- and postoperative plasma glutamine level was analysed with the paired-*t* test. Univariate and multivariate analysis were performed to evaluate the relations between plasma glutamine levels and LOS, rate of positive cultures and antibiotic usage.

A two-sided *P* value < 0.05 was considered significant. All tests of the data were performed using Statistical Package for the Social Sciences 21.0 (IBM, New York, NY, USA).

## Results

A total of 90 (17 female/73 male) patients were included. Sixty-two patients underwent CABG, eleven patients valve replacement (AVR/MVR), twenty-four patients CABG combined with valve replacement and three patients underwent another form of cardiovascular surgery. Patients characteristics are given in Table 1.

**Table 1:** Patients characteristics at admission on the ICU

Age (years)	68 (±8.3)
BMI	27.6 (±4.2)
ECC (minutes)	90 [67.8-118.3]
AOX (minutes)	59.5 [42-83.3]
EuroSCORE	4 [3-7]
APACHE IV score	46 (±12.8)
LOS ICU (days)	2.0 [2-2]
LOS Hospital (days)	5.6 [4-7]
Creatine kinase (U/L)	283 (±169)
Creatinine (μmol/L)	91 [71.8-91]
Phosfate (mmol/L)	0.64 (±0.27)
Magnesium (mmol/L)	1.40 (±0.30)

*BMI* Body mass index, *ECC* extracorporeal circulation, *AOX* aortic clamp time, *APACHE* Acute Physiology and Chronic Health Evaluation, *LOS* length of stay

Data are given as mean ± standard deviation or median and [IQR]

Plasma glutamine level pre-operative was 0.42 ± 0.10 mmol/l. Post-operative plasma glutamine level was 0.38 ± 0.09 mmol/l which was significantly lower compared to the pre-operative level (paired *t*-test *p* < 0.001). Before surgery 50 patients had a plasma glutamine level below 0.42 mmol/l (55%) en after surgery 65 patients had a plasma glutamine level below 0.42 mmol/l (72%).

There was no relation between duration of extracorporeal circulation or aortic occlusion time and changes in plasma glutamine levels (*p* = 0.88 en 0.95 respectively). The EuroSCORE in patients with a low glutamine was 4 [3–7] en with normal glutamine 4 [3–6], there was no significant difference in EuroSCORE between the groups (*p* = 0.36). There was a significant correlation between plasma glutamine levels post-operative and the LOS ICU (*p* = 0.01) and hospital (*p* < 0.001) but not with the pre-operative plasma glutamine level.

A positive culture was found in 14 patients during their post-operative admission; 8 airway, 3 urine, 2 wound and 1 blood. Five patients were treated with antibiotics. C-reactive Proteine (CRP) was only measured on indication, the highest mean CRP was 72 [47–117] U/l.

A logistic regression analysis showed a significant correlation between the presence of a positive culture and pre-operative plasma glutamine levels (*p* = 0.04). Also, the LOS ICU is associated with the presence of a positive culture during total hospital admission in a multivariate analysis (*p* =0.003). We did not find a relation between the % drop in plasma glutamine levels and the presence of an infection.

## Discussion

In the present study we found that plasma glutamine levels are significantly lower just after cardiac surgery compared to pre-operative levels. We did not find a relation between the decrease in plasma glutamine levels and the duration of extracorporeal circulation or aortic clamp time. Logistic regression analysis showed a correlation between pre-operative plasma glutamine levels and the presence of a positive culture after cardiac surgery.

It is not possible to make a distinction between the contribution of surgery or extracorporeal circulation on the effect in plasma glutamine levels in the present. In a study conducted in cardio-surgical patients the elevation in pro-inflammatory cytokines and acute-phase proteins did not differ between patients who underwent cardiac surgery with or without the use of cardiopulmonary bypass, and the authors concluded that the surgical trauma and reperfusion injury represented more immunological changes then the use of cardiopulmonary bypass [9, 10]. Plasma glutamine levels were measured in 9 patients following elective abdominal aortic aneurysm surgery, in this group plasma glutamine level significantly decreased in the hours following surgery and remained lowered until the end of the study, 168 h after start surgery [11]. So, it is more likely that the decrease in plasma glutamine levels is the result of surgery rather than the extracorporeal circulation.

We found a correlation between pre-operative plasma glutamine levels and the presence of a positive culture in the post-operative course. A positive culture was found in 16% of patients and 5.5% received antibiotics. In two large trials the reported percentage of postoperative infections after cardiac surgery varied from 9.5 to 17% [12, 13]. A post-operative infection can affect long term survival. In a large cohort of patients who underwent colon surgery the presence of any infectious complication was associated with a significantly worse long-term survival compared to patients without complications (HR:1.31, 95% CI1.21–1.42, *p* < 0.001) [14]. It is conceivable that postoperative infections after cardiac surgery also affects survival but this is not studied to this extent. Whether it is possible to identify patients with a higher risk of developing infections by measuring plasma glutamine level prior to surgery needs further study. Furthermore, can we improve outcome in patients with a low plasma glutamine level who will undergo elective surgery by supplementation of glutamine?

## Conclusion

Plasma glutamine levels are significantly lower just after cardiac surgery compared to pre-operative levels. There was a correlation between pre-operative plasma glutamine levels and the presence of a positive culture after cardiac surgery. Whether plasma glutamine levels can be used to identify patients with higher risk of infection needs further study.

## Abbreviations

AOX: Aortic clamp time
APACHE: Acute physiology and chronic health evaluation
BMI: Body mass index
CRP: C-reactive proteine
ECC: Extracorporeal circulation
ICU: Intensive care unit
LOS: Length of stay
